# A Correspondent

**Published:** 1896-02

**Authors:** 


### EDITORIAL.

A correspondent presents the following:

"DEAR SIR—I write to ask you a question. 'What is the best treatment for chemical abrasion of the upper incisors?' The teeth are abraded badly—the two centrals and laterals, commencing a little below the margin of the gum and extend about half way down the front. The abrasion at the upper edge, which is a little below the gum, is very deep so as to make almost a square shoulder, but decreases in depth as it goes down. It looks as if a half-round file had been drawn across the front. The depression has a perfectly smooth and polished surface, even more so than the enamel itself. The cuspids also are affected, but not so much, and in the first bicuspid you can see a trace of it. I

know it has not been caused by any mechanical action. The party is about forty-five years old, has taken the best care of his teeth and this has appeared in the last two or three years. What can be done for it? Will you please give me what information you can? It would confer a great favor on me as well as on the patient."

In reply to the above request, we may say that the affection you describe is often found, and usually on teeth of the better varieties, and even the best teeth are sometimes affected in this way; the inferior kinds of teeth are rarely if ever abraded in this manner. In regard to its cause there has been a number of theories advanced, but none of them fully established; these theories we need not discuss or even mention here; you have a case and wish to know what to do with it. In the first place, note the conditions of the mouth, the gums, the mucous membrane, the secretions, the saliva and mucus. If any of these are abnormal, determine the condition as accurately as possible; if the tissues are affected, restore to health by the appropriate treatment; if the secretions are vitiated markedly abnormal, they should be restored to a normal state, this can only be done by systemic treatment, which ought to be hygenic rather than by by medication; notwithstanding the latter will, in some cases, be required, and should be such as will act upon the secretory organs. In some cases changes may be brought about by simply hygenic means that will so change the condition of the mouth that the wasting of the teeth as above indicated will be retarded, in some cases altogether arrested. But so uncertain has all such treatment proved hitherto, that it cannot alone be relied upon generally for the arrest of the affection. Where grooves or pits of some considerable depth have been made, filling seem to be indispensable, and even where a large plain surface is wasting away by this process, putting on a covering of gold seems to be clearly indicated, as the most certain means of prompt arrest. We hope the time is near at hand when we will know more about this affection, and when we will be able to prevent its ravages by more certain means than we are now able to command.

